# Determining the Pathogenicity of Genetic Variants Associated with Cardiac Channelopathies

**DOI:** 10.1038/srep07953

**Published:** 2015-01-22

**Authors:** Oscar Campuzano, Catarina Allegue, Anna Fernandez, Anna Iglesias, Ramon Brugada

**Affiliations:** 1 Cardiovascular Genetics Center, Institut d'Investigació Biomèdica de Girona (IDIBGI) and Universitat de Girona (UdG), Girona, Spain; 2 Medical Science Department, School of Medicine, University of Girona, Girona, Spain; 3 Cardiology Service, Hospital Josep Trueta, Girona, Spain

## Abstract

Advancements in genetic screening have generated massive amounts of data on genetic variation; however, a lack of clear pathogenic stratification has left most variants classified as being of unknown significance. This is a critical limitation for translating genetic data into clinical practice. Genetic screening is currently recommended in the guidelines for diagnosis and treatment of cardiac channelopathies, which are major contributors to sudden cardiac death in young people. We propose to characterize the pathogenicity of genetic variants associated with cardiac channelopathies using a stratified scoring system. The development of this system was considered by using all of the tools currently available to define pathogenicity. The use of this scoring system could help clinicians to understand the limitations of genetic associations with a disease, and help them better define the role that genetics can have in their clinical routine.

## Sudden Cardiac Death

Sudden cardiac death (SCD) is defined as an unexpected and non-traumatic death of an individual who had been observed healthy in previous 6 hours of the death^1^. In western countries, SCD underlies 20% of total mortality. Although heart failure and coronary artery disease are the most prevalent substrates^2^, epidemiological studies indicate that monogenic syndromes –called inherited arrhythmogenic diseases- also plays an important role in cardiac electrical instability^3^. Thus, the inherited arrhythmogenic diseases have been defined broadly as 2 categories of pathologies: channelopathies and cardiomyopathies. Channelopathies are caused by pathogenic variations in genes encoding ion channels, and include Long QT Syndrome (LQTS), Brugada Syndrome (BrS), Short QT Syndrome (SQTS), and Catecholaminergic Polymorphic Ventricular Tachycardia (CPVT). Cardiomyopathies results from variations in genes encoding sarcomeric, cytoskeletal, and desmosomal proteins, and include Hypertrophic Cardiomyopathy (HCM), Arrhythmogenic Right Ventricular Cardiomyopathy (ARVC), and Dilated Cardiomyopathy (DCM).

Significant progress has been made in the identification of a genetic basis of SCD-related disorders. In particular, next-generation sequencing (NGS) of the human genome has increased dramatically the amount of genetic data available. Despite this advancement, 2 major genetic challenges in SCD-related diseases remain to be overcome^4^. First, **a more definite conclusion regarding pathogenicity of variations must be reached**. Most of the genetic data, even after bioinformatic evaluation, remains of uncertain significance. In addition, current international guidelines often focus on the prediction of disease outcomes and/or therapeutic measures only if patients carry a variant classified as pathogenic^5^. To our knowledge, few reports have defined pathogenicity and how to differentiate genetic causality from background noise. For example, a recent study indicated that 30% of disease-causing genetic variations cited in the literature are common polymorphisms or misinterpreted variants^6^. Thus, the second challenge is in **translating the genetic data into clinical practice**. Clinical interpretation of genetic data is becoming increasingly complex, and this is particularly true of SCD-related diseases, which are characterized by incomplete penetrance and variable expressivity and thereby complicate diagnosis and treatment. Current guidelines recommend adopting preventive measures depending on both clinical context and family history, but heritability remains of lower priority^5^.

If the genetic variations associated with SCD are to be of value in the clinic, their pathogenicity must be defined. A recent report describes a methodical assessment that decreases the average time to compile, analyse, and interpret variant data^7^. In this report, Duzkale et al. propose several items that should be analysed to classify the pathogenicity of each genetic variation. However, they do not achieve an accurate numeric scale of pathogenicity, thus the pathogenic classification remains ambiguous. Another recent report highlights the current challenges in investigating causality of human sequence variants, suggesting that each variation be assessed by combining different tools to determine disease contributions^8^. In the present report we propose a scale to stratify pathogenicity for genetic variations associated with channelopathies. We integrate multiple parameters to reach a more definite conclusion for use in the clinic.

## Channelopathies

Channelopathies are electrical disorders in structurally normal hearts caused by pathogenic variations in genes encoding cardiac ionic channels or regulatory proteins. These alterations modify the ionic balance of the electrical component of cardiac function and cause life-threatening arrhythmias^9^.

### Long QT syndrome

LQTS is a genetic disorder characterized by a prolongation of the QT interval on the ECG (QTc *>* 480 ms). The clinical presentation can be variable, ranging from asymptomatic to episodes of syncope and SCD due to ventricular tachyarrhythmia (*torsade de pointes*) in the setting of a structurally normal heart. The syncopal episode may be induced by exertion, and emotional or auditory stimuli. SCD is the first event in 5% of asymptomatic LQTS individuals. The estimated prevalence is 1/2500, but the penetrance of the disease is not 100%^10^. In 1993, the first clinical diagnosis of LQTS was published^11^. At present, genetic analyses are included in the scoring criteria for diagnosis^5^. Thus, individuals and family members at risk with a normal ECG may be identified through genetic testing. Currently, nearly 1200 pathogenic variations have been identified in 15 genes (*KCNQ1, KCNH2, SCN5A*, *ANK2, KCNE1, KCNE2, KCNJ2, CACNA1C, CAV3, CALM1, CALM2, SCN4B, AKAP9, SNTA1*, and *KCNJ5*)^12^. Combined, these genes contribute to approximately 80%–85% of all LQT cases. However, 70%–75% of LQT cases are attributable to pathogenic variations only in 3 genes, *KCNQ1* (LQT1), *KCNH2* (LQT2), and *SCN5A* (LQT3), with *KCNQ1* responsible for around 35% of cases. Genetic testing for LQTS has a major role in the diagnosis of index cases, risk stratification, family screening, and therapeutic strategies, but only for a small number of pathogenic variations^13^. Most identified genetic variations remain untranslated to the clinic.

### Brugada syndrome

BrS is an inherited disease described 20 years ago and characterized by an ECG pattern consisting of coved-type ST-segment elevation in atypical right-bundle branch block in leads V1 to V3 (type-1)^14^. BrS patients have an increased risk for SCD resulting from episodes of polymorphic ventricular tachyarrhythmia^15^. BrS accounts for 4%–12% of overall SCD and 20% of SCDs with normal hearts. The reported prevalence is 1 to 5/10000 in Europe and 12/10000 in Southeast Asia, with a ratio of 8:1 men:women affected. BrS typically affects young male adults (30–40 years old), and SCD typically occurs during sleep^15^. To date, more than 300 pathogenic variations in 16 genes (*SCN5A, GPD1-L, SCN1B, SCN2B, SCN3B, KCNE3, KCNE5, KCNJ8, KCND3, CACNA1C, CACNB2b, CACNA2D1, RANGRF, HCN4, SLMAP* and *TRPM4*) have been associated with BrS^16^. However, genetic testing only identifies the pathogenic cause in 35%–40% of clinically diagnosed cases of BrS. Approximately 25%–30% of diagnosed patients carry a pathogenic variation in the *SCN5A* gene^17^.

### Catecholaminergic Polymorphic Ventricular Tachycardia

CPVT is a familial disease characterized by severe arrhythmias under adrenergic stimulation, such as exercise or emotional stress. The resting ECG is usually normal, but exercise testing induces ventricular arrhythmia in 75%–100% of patients. In many cases, the first manifestation of CPVT is the death of the patient. CPVT is associated with high mortality (around 30% by the age of 30 years), and the estimated prevalence is 1/10000^18^. To date, more than 100 pathogenic variations have been identified in five genes, contributing to nearly 60% of all clinically diagnosed cases (*RYR2*, *CASQ2, KCNJ2, TRDN, CALM1*, and *CALM2*). The *RYR2* gene, encoding the ryanodine receptor (autosomal dominant), is responsible for nearly 50% of all cases^12^.

### Short QT syndrome

First reported in 2000^19^, SQTS is considered the most lethal channelopathy; it has a high familial incidence of palpitations or syncope, and SCD, typically during childhood, is often the only phenotypic manifestation. It is characterized by a short QT interval on the ECG (QTc < 325 ms) with a high sharp T wave^20^. The incidence and prevalence are difficult to determine due to limited data. So far, pathogenic variations have been reported in 4 genes (*KCNQ1, KCNH2, KCNJ2*, and *CACNA2D1*), and these account for nearly 50% of clinically diagnosed SQT cases^12^.

## Methods

In 2008, the American College of Medical Genetics and Genomics (ACMG) published guidelines for genetic variant interpretation^21^. However, most of the genetic tools outlined have been improved since 2008, and additional ones have been developed. Most of the current genetic data generated by high-throughput technologies has identified exonic *common variants* previously identified in the general population (minor allele frequency, MAF > 1%). Much of the remainder is classified as being of *unknown significance*, with only a small number of variants classified as *pathogenic*. To supplement the ACMG guidelines, we propose to incorporate new information to stratify the level of pathogenicity into 5 groups: pathogenic, probably pathogenic, unknown significance, probably non-pathogenic, and non-causal/benign.

**Figure 1:**
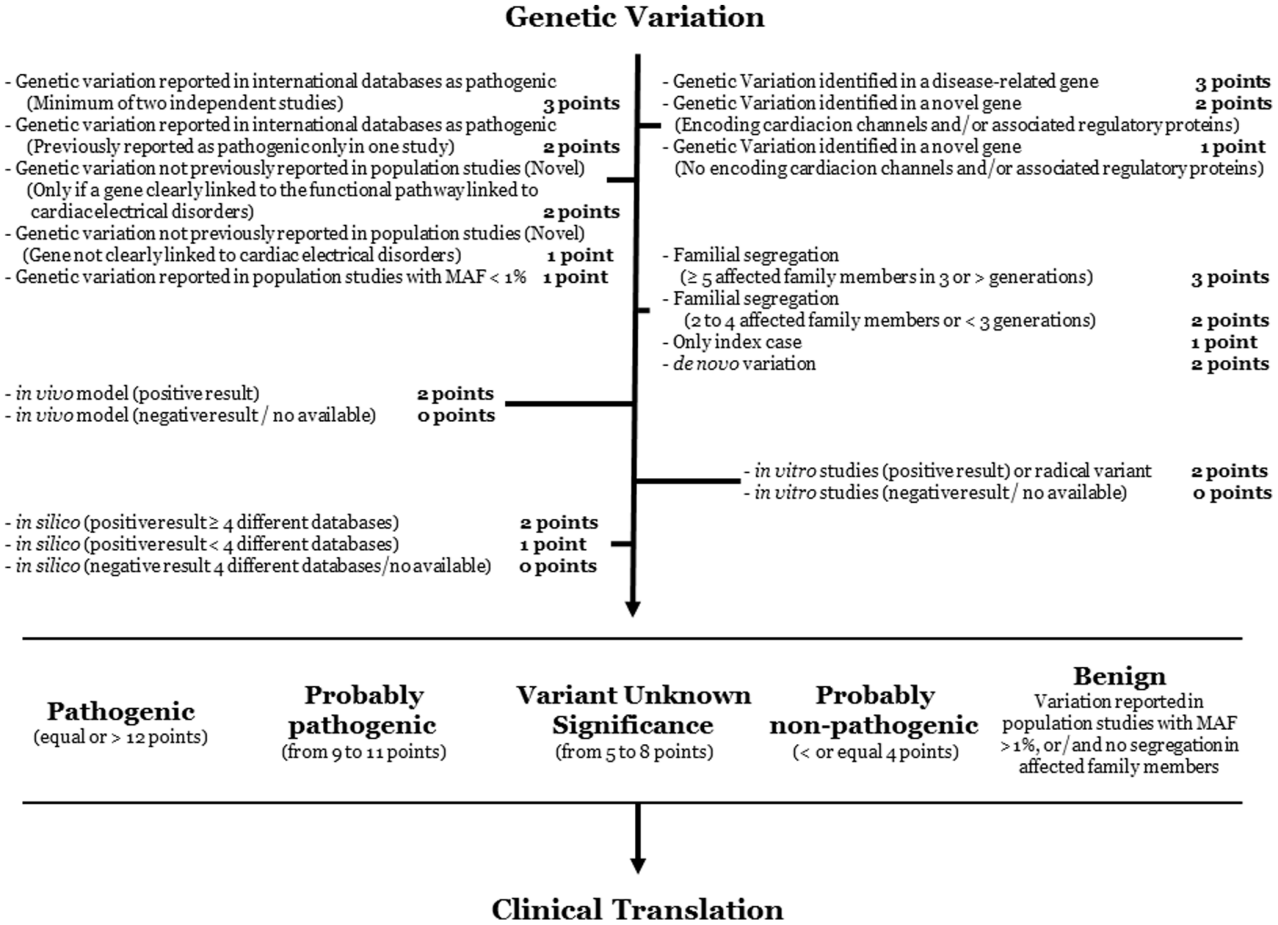
Scale classification workflow. The proposed items should be analysed to classify the variation (gene, variation, family segregation, *in vitro*, *in vivo*, and *in silico* studies). MAF: Minor Allele Frequency.

Our scale of pathogenicity (Figure 1) is based on the analysis of several items:- Clinical ascertainment (disease-associated gene or candidate gene) and determination of the genetic isoform in which the variation was identified;- Whether the variation has been previously identified in international databases;- Family segregation (identification of *de novo* variation may occur); and- *In vitro*, *in vivo*, and *in silico* evaluation.

We have categorized the scoring using a similar approach to that in the LQTS Diagnostic Criteria^11^. Our scores range from 0 to 15 points (Figure 1). Each item receives a score between 0 and 3 points, and the total of the assessed items determines whether the exonic genetic variation could be considered pathogenic (≥ 12 points), probably pathogenic (from 9 to 11 points), unknown significance (from 5 to 8 points), probably non-pathogenic (≤ 4 points), and benign. This last group comprises genetic variations considered as common in the global population (MAF > 1%). This fact implies that our score is useful only for non-silent variants identified in dominant disorders, in concordance to the diseases assessed in the present report. Finally, before final genetic interpretation of a variant, it is crucial to know the genetic isoform and tissue expression in order to perform an appropriate translation into clinical practice.

## Results

### Clinical ascertainment

The use of genetics as a clinical diagnostic tool must always consider the context of the clinical case. Therefore, genetics should complement, and never supplant, the clinical investigation. The advent of NGS, with its constant discovery of new associations of genes with diseases, prompts the need for the confirmation of pathogenicity in more patients and families. This is especially true in the study of SCD, in which segregation analyses are limited by the sizes of the families due to the sudden death of some affected individuals at a young age^22^. Without a clear clinical ascertainment, and a clear correlation between the disease and the genetic variant, causality cannot be conclusive.

Some proportion of the SCD cases arising from unknown genetic causes could be explained by pathogenic genetic variations in genes not yet associated with the disease. We hypothesize that the genes most likely to be associated with such cases are other genes encoding cardiac ion channels and/or regulatory proteins associated with them, independently of the cardiac channelopathy diagnosed. Recently, through the development and improvement of NGS technologies, several genes previously associated with only one channelopathy have been linked to other channelopathies; for example, the *SCN5A* gene contributes to both BrS and LQTS^23^.

- Genetic variation identified in a disease-related gene             3 points

- Genetic variation identified in a novel gene                  2 points

(Encoding cardiac ion channels and/or associated regulatory proteins)

- Genetic variation identified in a novel gene                  1 point

(Not encoding cardiac ion channels and/or associated regulatory proteins)

### Genetic databases

Several genetic databases are available, such as PubMed (www.ncbi.nlm.nih.gov/pubmed), the Exome Variant Server (http://evs.gs.washington.edu/EVS/), the 1000 Genomes Project (www.1000genomes.org), and the ClinSeq Project (www.genome.gov/clinseq). These databases catalog all the genetic data generated by published large-scale population studies, including pathogenic, potentially pathogenic, genetic variant of unknown significance (GVUS), and common genetic variations in the population. Other databases focus only on potential pathogenic variations [such as ClinVar (www.clinvar.com) and HGMD (www.hgmd.cf.ac.uk)]; however, caution should be exercised when regarding these classifications as not all characterizations are exhaustive and some of these classified variants could be benign. We believe that, for each reported pathogenic variation, the published source should be carefully analysed and interpreted. Importantly, only if a genetic variation has been reported as pathogenic in more than one study could it be suggested as damaging.

In our proposed stratification, this item determines if the variation is novel (never identified so far) or already reported. If reported, the variation could be considered *pathogenic, of unknown significance*, or *neutral/non-causal*. In addition, the genetic variation could be classified as a common (MAF > 1%) or rare (MAF < 1%) variation in the global population. (MAF < 1% does not imply certain pathogenicity).

- Genetic variation reported in international databases as pathogenic       3 points

(Minimum of two independent studies)

- Genetic variation reported in international databases as pathogenic       2 points

(Previously reported as pathogenic only in one study)

- Genetic variation not previously reported in population studies (Novel)      2 points

(Only if a gene is clearly linked to the functional pathway(s) in cardiac electrical disorders)

- Genetic variation not previously reported in population studies (Novel)      1 point

(Gene not clearly linked to cardiac electrical disorders)

- Genetic variation reported in population studies with MAF < 1%         1 point

One of the main limitations in genetic scenario is the previous report that a variant is pathogenic. Although there are several genetic variants already catalogued, recent whole exome/genome studies have demonstrated a large number of false-positive pathogenic variants that have been incorrectly classified^24^,^25^,^26^.

### Family segregation

Family history of syncope/SCD is a significant risk factor for other syncope/SCD in the family^5^. For this reason, familial genetic screening is recommended. However, the lack of information about pathogenicity of most known genetic variants implies that genetic screening does not always help to clarify the risk of syncope/SCD in a family.

In our proposed stratification, familial testing enables discernment of whether a variation is *de novo* or inherited. If *de novo*, the score should be high (2 points) because *de novo* variations are strongly associated with pathogenicity^27^. If a variant is suspected to be inherited, as many relatives as possible should be tested to clarify the role of variant. We believe that family segregation is the most important parameter that helps to clarify a variant's contribution, but should not be the only one investigated. For cases in which a clinically affected family member does not carry the potentially pathogenic variation identified in the index case (negative segregation), the genetic variation could be discarded, or at least ruled out as the main contributor to the disease, since it could be a modifier of the phenotype. If an asymptomatic family member carries the potentially pathogenic variation, the variation should not be discarded because of the possibility for incomplete penetrance. Therefore, in our stratification to determine pathogenicity, the presence of a minimum of 5 clinically affected carriers in at least 3 generations^28^ denotes high genotype-phenotype correlation (3 points). A lower number of affected carriers is given a score of 2 points; when the index case alone carries the variant, 1 point is assigned.

- Familial segregation (≥ 5 affected family members in 3 or more generations)   3 points

- Familial segregation (2 to 4 affected family members or < 3 generations)     2 points

- Only index case                             1 point

- *de novo* variation                             2 points

### *In vivo* studies

Experimental studies in animals are often used to recapitulate the phenomena underlying both normal and abnormal human biology. However, both economic and technological limitations prohibit the development of an animal model for each genetic variation. In our proposed scale, if an animal model is available for a variant and it recapitulates the human phenotype (positive result), 2 points are tallied due to the complexity of obtaining the data. We have not designated a score of 3 points in this category because the biological mechanisms exhibited in an animal model do not faithfully represent the mechanistic pathway(s) observed in humans. If an animal model is available but does not recapitulate the human phenotype (negative result) and/or no model is available, 0 points are given.

- *in vivo* model (positive result)                     2 points

- *in vivo* model (negative result/not available)               0 points

### *In vitro* studies

*In vitro* studies are one of the main experimental confirmations of pathogenicity in cardiac channelopathies. However, it is not feasible to perform *in vitro* studies to characterize each identified genetic variation. Even when available, some *in vitro* functional data not accurately reflect *in vivo* physiology^29^. This phenomenon occurs because *in vitro* studies are performed in heterologous systems that do not include all the necessary biological partners that modify the final phenotype.

Therefore, in our proposed stratification, if a cellular model is available for a variant and it mimics the biological mechanism(s) observed *in vivo* in humans (positive result), 2 points are tallied. In particular, “radical” genetic variations (*nonsense* and *indels*) have been assumed as pathogenic/deleterious because the protein will be altered; in consequence, 2 points are given in all these cases. As with the animal models, a score of 3 points is excluded from this category because *in vitro* studies cannot faithfully reflect the *in vivo* mechanistic pathway(s) in humans. If a cellular model is available but does not recapitulate the biological mechanism(s) observed *in vivo* in humans (negative result) or no model is available, 0 points are given.

- *in vitro* studies (positive result) or radical variation              2 points

- *in vitro* studies (negative result/not available)                0 points

### *In silico* studies

Several bioinformatic tools have been developed in recent years to predict pathogenicity [such as PROVEAN (http://provean.jcvi.org/index.php*)*, Condel ( http://bg.upf.edu/fannsdb/), SIFT (http://sift.jcvi.org/*)*, Polyphen2 (http://genetics.bwh.harvard.edu/pph2/*)*, Mutation Taster (www.mutationtaster.org*)*, and Mutation Assessor (www.mutationassessor.org)]. These computational tools predict the impact of the genetic variant on the gene/protein sequence, based on gene alteration, type of variation, protein structure, biochemical properties of amino acids, and even evolutionary sequence conservation between species. However, these items are not all included within one bioinformatic database. In addition, all genetic variations cannot be found in all bioinformatic databases. Consequently, splicing changes are identified in specific databases [such as Human Splicing Finder (http://www.umd.be/HSF/), NNSplice (www.fruitfly.org/seq_tools/splice.html), and GeneSplicer (www.ccb.jhu.edu/software/genesplicer)], just as intronic variations are identified in others [such as Alamut (www.interactive-biosoftware.com/alamut/doc/2.0/splicing.html)].

In our proposed scale, 0 points are assigned if bioinformatic analyses are not available for a variant in any of the computational databases, or, if they are available but all *in silico* analyses showed a neutral/benign prediction. To include the largest quantity of bioinformatic data possible, 2 points are assigned if more than 4 different databases agree on the predicted pathogenicity (deleterious, probably, and/or possibly pathogenic) and 1 point if less than 4 databases agree on the predicted pathogenicity of the variant. This last point includes, for example, 3 pathogenic and 1 benign, 2 pathogenic and 2 benign, or 4 benign predictions. This threshold is based on the fact that computational systems do not include all biological elements existing in humans. Regarding “radical” genetic variations, including *nonsense* genetic variations and frameshift/in-frame genetic variations, no database analyses them because it is assumed that the protein product will be altered; 2 points are given in all these cases. We do not assign a score 3 points because an *in silico* prediction cannot faithfully reflect a human mechanistic pathway, and the use of bioinformatic tools can produce erroneous conclusions regarding pathogenicity, as we reported recently^30^. A positive *in silico* result means that pathogenicity is predicted; in contrast, a negative result means that a neutral/benign variation is predicted.

- *in silico* (positive result in ≥ 4 different databases)               2 points

- *in silico* (positive result in < 4 different databases)               1 point

- *in silico* (negative result in 4 different databases/not available)          0 points

## Discussion

To validate our scale, we analysed several genetic variants localised in diverse genes associated and non-associated with different channelopathies (Table 1).

For LQTS, we analysed genetic variations localized in two different genes, *KCNQ1* and *KCNH2*. The first variation analysed was *KCNQ1*_p.R452W (c.1354C > T). This genetic variation has been previously associated with the disease^31^ -CM055335- (3 points), reported as pathogenic but only detected in the index case (2 point). No *in vivo* or *in vitro* studies were performed or identified. *In silico* analysis revealed four databases with damaging prediction (2 points). The total score of 8 points in this case indicates that the variant remains classified as a GVUS; this is in accordance with a recent report classifying *KCNQ1*_p.R452W as a variant of unknown significance^32^. Thus, this GVUS should be considered carefully in clinical practice. We believe that GVUS should be further analysed in relatives, since family segregation and genotype-phenotype correlation are required to clarify the pathogenic role of a variant.

The second variation was *KCNQ1*_p.Y315S (c.944A > C), previously associated with the disease^33^ -CM970823- (3 points) and reported as pathogenic in different studies (3 points). Familial segregation was detected in less than 4 relatives (2 points), and expression of the variant in rabbit heart produced transgenic rabbits with an LQT phenotype^34^ (2 points). *In vitro* analysis revealed a pathogenic role (2 points), and *in silico* analysis revealed 4 databases with damaging prediction (2 points). The total score of 14 points supports its classification as pathogenic in LQTS. In cases in which a pathogenic variation has been identified, current clinical/genetic guidelines should be followed both in the index case and in family members.

In *KCNH2*, also associated with LQTS, we analysed 2 genetic variations. The first one was *KCNH2*_p.E58A (c.173A > C), which was previously associated with the disease^35^ -CM057129- (3 points), reported as pathogenic only in one study (2 points), and was detected only in the index case (1 point). No *in vivo* or *in vitro* studies were identified, and *in silico* analysis revealed 3 databases with pathogenic prediction (1 point). The total score of 7 points indicates that the variant is likely a GVUS. Hence, further studies such as family segregation and *in vivo* and/or *in vitro* analyses should be performed to clarify its role in LQTS. The last variation associated with LQTS that we analysed was *KCNH*2_p.G628S (c.1882G > A), which was reported to be associated with the disease^36^ -CM950710- (3 points) and as pathogenic in more than one study (2 points). Familial segregation was identified in < 4 relatives (2 points), and expression in rabbit heart produced transgenic rabbits with an LQT phenotype^34^ (2 points). *In vitro* analysis revealed a pathogenic role (2 points), and *in silico* analysis revealed 4 databases with damaging prediction (2 points). A total score of 13 points supports the classification of this variant as pathogenic in LQTS.

For CPVT, 2 variations were analysed. The first one was *RyR2*_p.R1013Q (c.3038G > A), previously associated with the disease^37^ -CM097930- (3 points) reported as pathogenic but only detected in the index case (2 point). Familial segregation was identified only in the index case (1 point) and no *in vivo* or *in vitr*o studies were performed. *In silico* analysis revealed two databases with damaging prediction (1 point). A score of 7 points suggests classification as a GVUS, in accordance with a recent report^32^. The second was *RyR2*_p.V2475F (c.7423G > T), previously reported associated with the disease^37^ -CM097960- (3 points), and with a deleterious effect in more than one study (3 points). Familial segregation was identified only in the index case (1 point), and *in vivo* studies yielded a positive result (2 points)^38^. *In vitro* studies also showed a positive effect (2 points), and *in silico* analysis revealed 4 databases with damaging prediction (2 points). The total score of 13 points supports classification of this variant as pathogenic in CPVT.

Finally, three variations were analysed for BrS. The first one was *SCN5A*_p.V1340I (c.4018G > A), previously associated with the disease^17^ -CM100703- (3 points), and with a deleterious effect reported in only one publication (2 points). It was identified only in the index case (2 points), and no *in vivo* studies were performed. *In vitro* studies showed a functional effect (2 points), and *in silico* analysis revealed three databases with damaging prediction (1 point). A score of 9 points indicates classification of this variant as probably pathogenic. Therefore, further studies should be performed to clarify its role in BrS.

The second variation analysed in BrS was *SCN5*A_p.R104Q (c.311G > A), previously reported associated with the disease^39^ -CM014904- (3 points), and with a deleterious effect in some studies (3 points). Positive familial segregation was identified in less than 4 relatives (2 points) but no *in vivo* studies were performed. *In vitro* studies showed a functional effect (2 points), and *in silico* analysis revealed four databases with damaging prediction (2 points). The total score of 12 points indicates pathogenicity.

The last variation showing a disease-association gene was *SCN5A*_p.N70K (c.210T > G), previously reported to be associated with the disease^17^ -CM100623- (3 points), and with a deleterious effect but only in one report (2 points). It was identified only in the index case (1 point), and no *in vivo* or *in vitro* studies were performed. *In silico* analysis revealed two databases with damaging prediction (1 point). The total score of 7 points supports classification of this variant as a GVUS.

To further assess our scale of pathogenicity, we include some variation in genes showing a reported association with the disease. Hence, we analyze *PKP2*_p.Q62K in a case of suspicious BrS. The genetic variation is located in a novel gene which encodes a desmosomal protein (1 point). The variation is already reported only in one study as pathogenic (associated with arrhythmogenic cardiomyopathy) (2 points), but only identified in the index case (1 point). Neither *in vivo* nor *in vitro* studies were performed (0 points respectively), and *in silico* analysis revealed two databases with damaging prediction (1 point). The total score of 5 points indicates GVUS, in concordance to published studies^40^.

We also analyzed *SCN1Bb*_p.P213T in a case of suspicious LQT. The genetic variation is located in a novel gene which encodes an ion channel regulatory protein (2 points). The variation is novel but the gene is linked to functional pathways of cardiac electrical disorders (2 points). No segregation was reported (1 point), and no *in vivo* studies are available (0 points). Both i*n vitro* and *in silico* studies showed positive results (2 points). Therefore, total score of 9 points indicates probably pathogenic, as suggested in a recent publication^41^.

## Conclusions

Determining the pathogenic role of genetic variants in channelopathies associated with SCD is necessary for improved clinical diagnosis and therapy of inherited arrhythmogenic diseases. To date, most identified genetic variants are considered rare variants of ambiguous clinical significance.

In the present report we propose a first approach in stratification of pathogenicity for genetic variations associated with channelopathies. Using several genetic tools available in the biomedical field, we propose a stratified scale of pathogenicity for variants identified in genes associated with SCD. We bring together multiple well accepted parameters that should be used together to reach a more definite clinical conclusion. We believe that this stratification may help determine the pathogenicity of genetic variants and will help clinicians understand the limitations of genetics and to better decide on therapeutic measures to prevent syncopal episodes and SCD.

## Limitations

However, some limitations have to be acknowledged in the use of this approach. First, we propose the scale using several tools, but not all tools are available for each variant. It is not prudent to classify a variant using only one of these tools. Second, as genetic information is progressively becoming available, it is possible that new genetic/clinical data can change the pathogenicity level in a near future. We recommend reassessing the results on a regular basis. Finally, expertise is crucial in the handling of the databases and interpretation of the information. Thus, translation into clinical practice should be performed by consensus of a group of experts in different clinical and basic disciplines.

## Author Contributions

O.C., C.A., A.I. wrote the manuscript. O.C., A.F., R.B. designed the study. All authors reviewed the manuscript.

## Figures and Tables

**Table 1:** Examples of the stratified scoring system. LQT: Long QT Syndrome. BrS: Brugada Syndrome. CPVT: Catecholaminergic Polymorphic Ventricular Tachycardia. GVUS: Genetic Variant Unknown Significance.

Disease	Variation	Isoform	Disease-associated	Reported	Familial Segregation	*In vivo*	*In vitro*	*In silico*	Score	Classification of Variant
LQT	*KCNQ1*_p.R452W	NM_000218.2	3	2	1	0	0	2	8	GVUS
LQT	*KCNQ1*_p.Y315S	NM_000218.2	3	3	2	2	2	2	14	Pathogenic
LQT	*KCNH2*_p.E58A	NM_000238.3	3	2	1	0	0	1	7	GVUS
LQT	*KCNH2*_p.G628S	NM_000238.3	3	2	2	2	2	2	13	Pathogenic
CPVT	*RyR2*_p.R1013Q	NM_001035.2	3	2	1	0	0	1	7	GVUS
CPVT	*RyR2*_p. V2475F	NM_001035.2	3	3	1	2	2	2	13	Pathogenic
BrS	*SCN5A*_p.V1340I	NM_198056.2	3	2	1	0	2	1	9	Probably Pathogenic
BrS	*SCN5A*_p.R104Q	NM_198056.2	3	3	2	0	2	2	12	Pathogenic
BrS	*SCN5A*_p.N70K	NM_198056.2	3	1	1	0	0	1	6	GVUS
BrS	*PKP2_*p.Q62K	NM_004572.3	1	2	1	0	0	1	5	GVUS
LQT	*SCN1Bb_*p.P213T	NM_199037.3	2	2	1	0	2	2	9	Probably Pathogenic
